# Non-invasive ventilation for general paediatricians

**DOI:** 10.1007/s00431-026-07489-y

**Published:** 2026-10-26

**Authors:** Manu Sundaram, Alberto Medina, Marti Pons-Òdena

**Affiliations:** 1 Pediatric Medicine, Critical Care Division, Sidra Medicine, Doha, Qatar; 2 Department of Clinical Pediatrics, Weill Cornell Medicine-Qatar, Doha, Qatar; 3 Pediatric Intensive Care Unit, Hospital Universitario Central de Asturias, Oviedo, Spain; 4 Pediatric Intensive Care Unit, Hospital Sant Joan de Déu, Barcelona, Spain

**Keywords:** Non-invasive ventilation, High-flow nasal cannula, CPAP, BiPAP, ICEMAN, Paediatric acute respiratory failure

## Abstract

Over the past two decades, non-invasive ventilation (NIV) has transitioned from being primarily used in specialist intensive care units to becoming a standard intervention in high dependency units, general paediatric units, emergency departments, and increasingly, patients’ homes. Despite this expansion, many general paediatricians still perceive NIV as outside their primary expertise. With this review, we aim to address that gap and equip general paediatricians with the knowledge and confidence to appropriately initiate NIV, monitor its effectiveness, and recognise when escalation of care is required. We narratively reviewed the physiological rationale for non-invasive respiratory support (NRS), including high-flow nasal cannula (HFNC) and the two principal modalities of NIV—continuous positive airway pressure (CPAP) and bilevel positive airway pressure (BiPAP)—and summarised disease-specific evidence from recent literature, including randomised trials, systematic reviews, and international consensus statements. This is a narrative rather than a systematic review: we searched PubMed/MEDLINE and Paediatric Respiratory Reviews for English-language publications from January 2018 to June 2026 using the terms “non-invasive ventilation”, “high-flow nasal cannula”, “CPAP”, “BiPAP”, and “children”, supplemented by references already known to the authors, and selected articles for relevance and recency, prioritising randomised trials, systematic reviews, and consensus statements. HFNC is an oxygenation device rather than a ventilatory device, and its mechanism, clinical applications, and therapeutic goals differ from CPAP and BiPAP. We introduce the Indications, Contraindications, Equipment, Mode and settings, Assessment and monitoring, Next steps (ICEMAN) framework as a practical tool for structured and safe initiation of NIV in routine clinical practice, and apply it across bronchiolitis, acute severe asthma, pneumonia and hypoxaemic respiratory failure, neuromuscular disease, and obstructive sleep apnoea. *Conclusion*:  The essential requirements to initiate NIV outside the PICU are well-trained staff, appropriate monitoring, a reliable oxygen supply, and a clear plan for escalation to a higher level of care. Selection of patients for NIV outside the PICU requires careful attention to indication, contraindications, equipment, monitoring, and escalation pathway by using frameworks like ICEMAN.

**Table:** 

**What is Known:** • *HFNC, CPAP, and BiPAP are established, physiologically distinct respiratory therapies now used well beyond the PICU.*
**What is New:** •* The ICEMAN framework, updated with PALICC-2 ARDS criteria, gives non-specialists a structured, teachable approach to safe NIV initiation.*

**What is Known:**

• *HFNC, CPAP, and BiPAP are established, physiologically distinct respiratory therapies now used well beyond the PICU.*

**What is New:**

•* The ICEMAN framework, updated with PALICC-2 ARDS criteria, gives non-specialists a structured, teachable approach to safe NIV initiation.*

## Introduction

We have all spent many nights on call observing children struggling to breathe. The clinical signs—nasal flaring, tracheal tug, and paradoxical chest movement—are deeply unsettling. Conversely, there is significant professional satisfaction in applying an appropriate interface, adjusting the settings, and witnessing rapid improvement in respiratory effort. This exemplifies the effectiveness of non-invasive ventilation (NIV) when applied correctly.

NIV—the delivery of respiratory support through a non-invasive interface rather than an endotracheal tube—has transformed paediatric respiratory medicine. We use it acutely for respiratory failure, chronically for neuromuscular and sleep disorders, and increasingly as a bridge to avoid intubation in patients for whom intubation carries its own high risks. In a 2011 survey of Italian paediatric intensive care units (PICUs), NIV was recorded in 8.8% of all admissions and in 15.3% of non-ventilated admissions [1]. More recent UK PICANet registry data reinforce a rising trend in acute NIV use: an analysis of 151,128 consecutive PICU admissions across 31 UK PICUs between 2007 and 2014 found that NIV was used as first-line therapy in 32% of ventilated patients, and that an NIV-first strategy was associated with a 3.1% absolute reduction in mortality compared with invasive ventilation first [34].

Two new narrative review series published in 2025 in Paediatric Respiratory Reviews confirm that the evidence base is expanding rapidly, though much of it is extrapolated from adult studies or observational paediatric data [2, 3]. For the safe and structured initiation of acute NIV, Indications, Contraindications, Equipment, Mode and settings, As-sessment and monitoring, Next steps (ICEMAN) was developed by Medina, Pons-Òdena, and Sundaram through the ESPNIC FAST-NIVT project [4]. The acronym provides a practical framework that non-specialist paediatricians can use with confidence.

In this review, we address each component of the ICEMAN framework within the context of common paediatric presentations, whilst integrating the most recent clinical evidence. We intentionally use the first-person perspective to foster a collegial and peer-oriented discourse.

## Why NRS works in children: the physiological case

Understanding the physiological basis of non-invasive respiratory support (NRS) helps select appropriate modalities and minimises the risk of misapplication. Physiologically, paediatric respiratory failure differs from adult respiratory failure and requires a tailored approach.

Children have narrow airways. According to Poiseuille’s law, which describes laminar flow through a tube, airway resistance is inversely proportional to airway radius. This means that even modest mucosal swelling—as seen in bronchiolitis or croup—results in disproportionately large increases in work of breathing.

High-flow nasal cannula (HFNC) therapy washes out the nasopharyngeal dead space and delivers warm, humidified gas, optimising mucociliary function. It also generates a variable, flow-dependent positive end-expiratory pressure (PEEP) of 2–5 cm H₂O. HFNC delivers heated and humidified gas at flow rates exceeding the patient’s peak inspiratory demand—typically 1.5–2 L/kg/min in infants (up to 8 L/min for those under 12 months in most protocols) and 50–60 L/min in older children and adolescents [5]. The nasal cannula is positioned just inside the nares, offering comfort, ease of application, and high tolerability. This option allows parents to hold and feed their child and enables nursing staff to perform suctioning without difficulty. For many general paediatricians, HFNC is the most accessible initial form of NRS. Currently, the best evidence supports its use in patients with bronchiolitis who fail low-flow oxygenation in the general ward. In one large a randomized controlled trial (RCT), most infants avoided escalation of care regardless of allocated therapy (88% with HFNC versus 77% with standard low-flow oxygen) [6], with a further RCT reporting comparable findings [7], though it remains unclear whether HFNC itself prevents PICU admission.

The infant chest wall is also highly compliant—the ribs are cartilaginous, the intercostal muscles are immature, and the accessory muscles fatigue quickly. When respiratory load increases, infants generate large negative intrapleural pressures to expand their lungs. But the compliant chest wall collapses inward rather than expanding outward. This conflicting movement requires enormous effort and dramatically reduces breathing efficiency.

Positive end-expiratory pressure (PEEP), delivered by continuous positive airway pressure (CPAP), counteracts this directly by delivering a constant pressure throughout the entire respiratory cycle—typically 5–8 cmH₂O—splinting the chest wall open, recruiting atelectatic alveoli, and improving functional residual capacity (FRC). CPAP does not provide active inspiratory support. The primary benefits occur at end-expiration, including alveolar recruitment, prevention of airway collapse, improved compliance, and reduced work of breathing. CPAP is most frequently utilised in cases of alveolar collapse or upper airway obstruction, such as severe pneumonia, post-extubation support, apnoea of prematurity, and obstructive sleep apnoea (OSA).

It is important to note that CPAP commonly does not correct hypercapnia. If a child demonstrates rising CO₂ levels and there are concerns for respiratory pump failure, CPAP alone is insufficient—except in mild-to-moderate bronchiolitis. In this setting, CO₂ retention more often reflects increased dead-space ventilation and fatigue-related hypoventilation secondary to air trapping rather than primary respiratory pump failure. By splinting the airway and reducing air trapping CPAP can indirectly improve alveolar ventilation and CO₂ clearance even though it provides no active inspiratory support. Escalation to bilevel positive airway pressure (BiPAP) is required in most of these cases. Some infants with severe bronchiolitis can present with marked chest wall retraction and hypoxaemia due to collapse. CPAP application frequently results in a rapid reduction in breathing work, alveolar recruitment, and improvement in oxygenation. This illustrates how PEEP can restore more effective gas exchange.

BiPAP works on a third problem: the failing respiratory pump. When the child cannot sustain the muscular effort to generate adequate tidal volumes—because of neuromuscular weakness, severe obstructive load, or sheer exhaustion—they need active inspiratory augmentation. BiPAP cycles between two pressures: a higher inspiratory positive airway pressure (IPAP) and a lower expiratory positive airway pressure (EPAP). The EPAP does the same job as CPAP. However, the difference between IPAP and EPAP lies in the pressure support: EPAP actively inflates the chest with each breath. That pressure support is what BiPAP offers, which CPAP and HFNC simply do not. This makes BiPAP essential in hypoventilation syndromes and the sickest patients. We typically start with IPAP 10–12 cm H₂O and EPAP 4–6 cm H₂O, then adjust upward based on clinical response and, where available, transcutaneous CO₂ monitoring.

A back-up rate can be programmed to deliver mandatory breaths when the patient’s own rate falls below a threshold—essential for children with apnoeas, those unable to activate the trigger, or those experiencing severe neuromuscular weakness. BiPAP with a back-up rate is, in effect, non-invasive pressure-controlled ventilation and forms the foundation of long-term home NIV in children with progressive respiratory muscle failure.

## Comparing the three options

Table 1 translates the physiological principles above into a single, quick bedside reference, summarising how HFNC, CPAP, and BiPAP differ in mechanism, typical starting settings, indications, contraindications, and monitoring priorities (Table 1).

**Table 1:** Comparison of high-flow nasal cannula (*HFNC*), continuous positive airway pressure (*CPAP*), and bilevel positive airway pressure (*BiPAP*): mechanisms, settings, indications, and monitoring

Feature	HFNC	CPAP	BiPAP
Primary mechanism	Dead-space washout; flow-dependent PEEP 2–5 cm H₂O	Fixed PEEP; alveolar recruitment; improved FRC	PEEP + active inspiratory support (pressure support = IPAP − EPAP)
Corrects hypercapnia	Minimally	No (except mild bronchiolitis)	Yes — primary indication
Interface	Nasal cannula	Nasal or oronasal mask	Nasal or oronasal mask
Child tolerability	Excellent	Moderate	Moderate
Typical starting settings	1–2 L/kg/min (infants); up to 60 L/min (adolescents); FiO₂ titrated to SpO₂ 92–97%	5–7 cm H₂O; titrate by 1–2 cm H₂O increments; max 8–10 cm H₂O	IPAP 10–12, EPAP 4–6 cm H₂O; back-up rate optional; FiO₂ titrated to SpO₂ 92–97%
Key acute indications	Bronchiolitis; mild–moderate ARF; post-extubation step-down	Bronchiolitis; alveolar collapse; severe pneumonia; post-extubation; OSA	Hypercapnic/type 2 RF; severe asthma; neuromuscular disease; ARDS (limited trial)
Key chronic indications	Not used for long-term NIV	OSA (first-line where AT has failed)	NMD with nocturnal hypoventilation; central hypoventilation; CLD
Where can it be started?	General ward, ED, PICU; district-level hospitals	Monitored ward, HDU, PICU	HDU, PICU (acute); community/home (long-term)
Contraindicated if…	Active haemodynamic compromise; immediate intubation required	Inability to protect the airway	Haemodynamic instability; inability to protect airway; severe facial trauma
Monitoring priority	Work of breathing (RR, recession), SpO₂/FiO₂ ratio, HR	As HFNC + capillary CO₂ at 1–2 h	As CPAP + transcutaneous CO₂ preferred; blood gas at initiation and 1–2 h
Failure threshold (act now)	FiO₂ > 50% after a couple of hours of HFNC, or S/F ratio ≤ 250 despite optimisation (PALICC-2 NIV-PARDS threshold) [17]	As HFNC	FiO₂ above 60% after 4-h trial of BiPAP, worsening WOB and blood gas, or S/F ratio ≤ 250 despite optimisation (PALICC-2 NIV-PARDS threshold) [17]

*ARF*, acute respiratory failure; *AT*, adenotonsillectomy; *CLD*, chronic lung disease; *EPAP*, expiratory positive airway pressure;* FRC*, functional residual capacity;* HFNC*, high-flow nasal cannula;* IPAP*, inspiratory positive airway pressure;* NMD*, neuromuscular disease; *OSA*, obstructive sleep apnoea; *PEEP*, positive end-expiratory pressure; *RF*, respiratory failure; *WOB*, work of breathing

The FiO₂/time-based failure thresholds shown for HFNC and BiPAP reflect pragmatic, expert-consensus practice rather than a specific validated trial-derived rule for each numeric cut-off; the S/F ratio threshold of ≤ 250 is, by contrast, taken directly from the PALICC-2 NIV-PARDS definition [17], similar in spirit to the RCT-informed PALICC-2 4-h rule applied in ARDS (Sect. “Pneumonia and hypoxaemic respiratory failure”)

## Disease-specific evidence

### Bronchiolitis

Bronchiolitis is the most common acute indication for NIV in young children, and the area where the evidence is most dynamic. The disease causes small airway inflammation and oedema, air trapping, ventilation-perfusion mismatch, and rapidly progressive fatigue, particularly in infants under 3 months, ex-premature infants, and those with congenital heart disease or chronic lung disease.

We now have randomised trial data comparing HFNC and BiPAP in this setting. A 2024 trial from Brazil found that HFNC was noninferior to BiPAP for preventing intubation in infants under 2 years with mild-to-moderate respiratory failure due to bronchiolitis [8]. However, a 3-year RCT from a tertiary PICU found that CPAP or nasal positive-pressure ventilation was significantly superior to HFNC in preventing treatment failure (70.4% versus 50.7%, *p* = 0.001), with switching to CPAP after HFNC failure avoiding intubation in 54% of cases [9]. This apparent conflict likely reflects differences in study population and setting. The non-inferiority trial enrolled infants with mild-to-moderate disease across mixed ward and PICU settings. The superiority trial was conducted in a single tertiary PICU in a more severely affected population, in whom the additional pressure support of CPAP/NPPV may confer greater benefit.

The ongoing BACHb trial—a multicentre UK RCT comparing standard oxygen therapy, HFNC, and CPAP in bronchiolitis—will provide the most definitive guidance yet [10]. Until those data arrive, a reasonable approach is to start with low-flow oxygen therapy (LFOT) and, in case of failure, escalate to HFNC on the floor. Escalation to CPAP on the high dependency unit (HDU) or floor should be governed by institutional policies.

The key clinical signal we watch for is the work of breathing, not just saturations or the SpO₂/FiO₂ ratio. Although SpO₂/FiO₂ (S/F) calculations are recommended with HFNC, it is important to note that the actual alveolar FiO₂ cannot be determined with HFNC [11, 12], though interface design and monitoring technology have evolved substantially since these estimates were derived. Failure criteria described by Schibler et al.—heart rate > 150 and respiratory rate > 50 at 2 h—remain clinically useful bedside predictors of need for escalation [13], although this cut-off has not been re-validated in more recent bronchiolitis cohorts. Several clinical scales, including the Modified Wood-Downes Clinical Asthma Score (M-WCAS), Respiratory Severity Score, Bronchiolitis Score of Sant Joan de Déu (BROSJOD), and Tal-modified score, have been used to monitor patients with bronchiolitis. The best scale to confirm escalation (hospital admission) is the BROSJOD scale, as the likelihood ratio (LR) and weight of evidence (WoE) demonstrated (LR-, −2.70; WoE+, 8.54) [14].

### Acute severe asthma

In asthma, there is dynamic air trapping. In children with moderate to severe asthma, if they are displaying signs of clinical deterioration, NIV can be tried prior to intubation. The priority is to reduce air trapping by allowing adequate exhalation. External PEEP, if used, should generally be low (0–5 cm H₂O) and individualised based on the patient’s intrinsic PEEP, respiratory mechanics, and ventilator synchrony. NIV should never be used to postpone necessary intubation.

Concomitant use of vibrating-mesh nebulisation during NIV increases drug deposition, potentially improving efficacy. A 2025 systematic review and meta-analysis concludes that HFNC does not demonstrate superior outcomes compared with conventional oxygen therapy or other non-invasive ventilation in children with severe asthma [15]. In our clinical practice, we use BiPAP for children with acute severe asthma who do not respond to maximal medical therapy, typically initiated at IPAP 12–14 cm H₂O and EPAP 5–7 cm H₂O, then titrated carefully.

### Pneumonia and hypoxaemic respiratory failure

Pneumonia is common and heterogeneous—the evidence for NIV is less robust here than for bronchiolitis, partly because the causative organism, the child’s immune status, the lobar distribution of disease, and the degree of hypoxaemia all vary widely. A 2021 Bayesian network meta-analysis—still the most thorough comparison of NIV modalities in children with acute lower respiratory infection—suggested BiPAP was associated with the greatest reduction in intubation rate and mortality, though confidence intervals were wide [16].

Doctors must identify patients with acute respiratory distress syndrome (ARDS) early, since NIV failure is markedly higher in this group than in other causes of respiratory failure (50% versus 15%) [18], an estimate that predates the Second Pediatric Acute Lung Injury Consensus Conference (PALICC-2) diagnostic criteria [17] and has not been re-validated using the updated definitions. The PALICC-2 recommends that moderate-to-severe paediatric acute respiratory distress syndrome (PARDS) be managed with invasive ventilation, allowing only a brief, time-limited trial of HFNC or NIV—no longer than 4 h—before escalation, with ARDS severity stratified using the SpO₂/FiO₂ (S/F) ratio [17]. This is consistent with observational data showing that a substantial proportion of children with PARDS are already managed with NIV in current practice [19]. It is essential to counsel families early about the prognosis, as a patient failing on NIV will require intubation; these discussions should occur beforehand rather than during acute deterioration.

### Neuromuscular disease

Children with Duchenne muscular dystrophy (DMD), spinal muscular atrophy (SMA), congenital myopathies, and other progressive neuromuscular conditions will inevitably develop respiratory muscle weakness. Sleep-related hypoventilation arrives before daytime symptoms, often silently. A 2023 review from the University of California highlighted that sleep-disordered breathing may be the first clinically detectable manifestation of neuromuscular weakness [20]. This is why, in our own institutional practice rather than as a universally published standard, we perform annual polysomnography in all children with progressive conditions and not just those who are symptomatic. General paediatricians will typically undertake this surveillance jointly with, or under the guidance of, a specialist neuromuscular or sleep service.

NIV corrects nocturnal hypoventilation, improves sleep architecture, preserves daytime lung function, reduces hospitalisation rates, and prolongs survival—particularly in DMD and SMA type 1 [21]. We start NIV when there is evidence of sleep-related hypoventilation (peak TcCO₂ > 6.5 kPa, oxygen desaturation index > 10/h), or when there are clear symptoms of sleep-disordered breathing; we do not wait for daytime hypercapnia, since by that point the respiratory reserve is critically depleted [23].

A key finding from recent literature is that mortality in neuromuscular disease may precede a decline in NIV adherence, rather than result from suboptimal ventilator settings [22]. Therefore, reviewing device download data—including usage time and leak data—at every clinic visit is a critical, potentially life-saving practice rather than a mere administrative task, though in general paediatric practice this review is usually undertaken jointly with, or under the guidance of, a specialist home-NIV team rather than independently.

### Obstructive sleep apnoea

Patient groups at risk of OSA include children with obesity, Down syndrome, and craniofacial conditions. CPAP is the second-line treatment for OSA in children where adenotonsillectomy has failed, is contraindicated, or is insufficient. BiPAP is preferred when there is concurrent hypoventilation.

NIV adherence in children with OSA is notoriously difficult. It requires a genuinely child-centred approach: careful interface fitting (often over multiple clinic visits), family education, behavioural strategies, and ongoing support. The ongoing UK SPIRITUS study is evaluating a structured toolkit of interventions to improve adherence to NIV in children—a critically needed evidence base [24].

## The ICEMAN framework: a structured approach to safe NIV initiation

We developed the ICEMAN framework through our involvement with the ESPNIC FAST-NIVT teaching programme, a global initiative to promote evidence-based NIV training across paediatric and neonatal intensive care [4]. The acronym was developed by Medina, Pons-Òdena, and Sundaram as a practical clinical scaffold for judicious patient selection, equipment choice, settings optimisation, and failure recognition [4]. We have found it helpful for teaching, clinical handovers, and structuring our own thinking at the bedside.

**Fig. 1:**
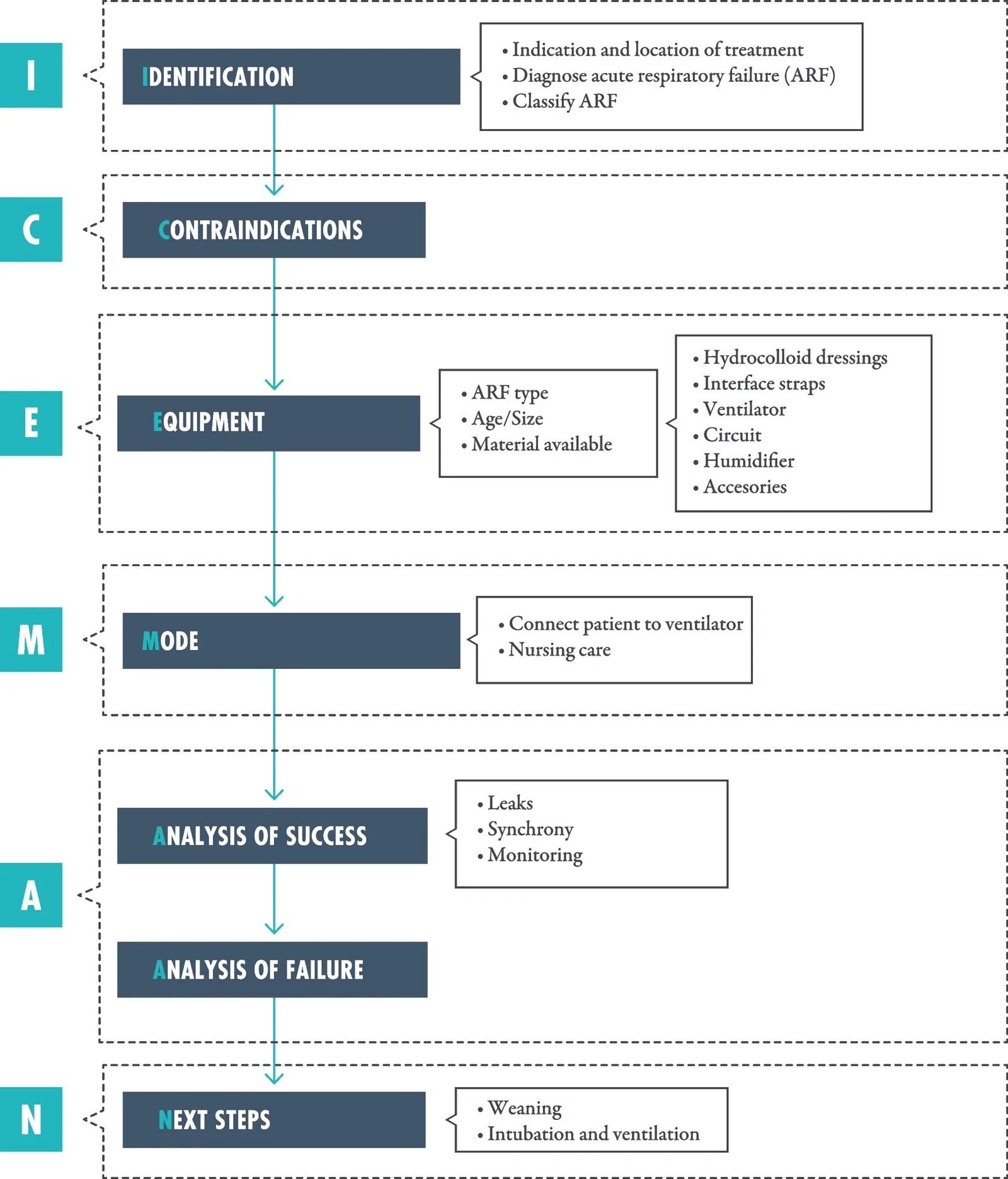
The ICEMAN framework for initiating and managing non-invasive ventilation in children. ARF, acute respiratory failure; ARDS, acute respiratory distress syndrome; NIV, non-invasive ventilation; CPAP, continuous positive airway pressure; BiPAP, bilevel positive airway pressure. Reproduction with permission: *Handbook of Pediatric and Neonatal Mechanical Ventilation (SECIP and ESPNIC)*. 2nd ed. Medina Villanueva A et al. (Eds.). *Las Palmas de Gran Canaria* (Spain): Tesela Ediciones. 2021 [25]

ICEMAN stands for Indications, Contraindications, Equipment, Mode and settings, Assessment and monitoring, and Next steps (failure and weaning). Figure 1 depicts the framework schematically, and Tables 2, 3, 4, and 5 set out each element in turn.

**Table 2:** ICEMAN framework — indications and contraindications

Type 1 respiratory failure — hypoxaemic	Type 2 respiratory failure — hypercapnic
Ventilation-perfusion mismatch Bronchiolitis Pneumonia Asthma Acute pulmonary oedema	Pump failure or restrictive pathology Neuromuscular disease (DMD, SMA, congenital myopathies) Chronic lung disease — BPD, cystic fibrosis Central hypoventilation syndromes May present with a type 1 picture if intercurrent infection
ARDS identification (PALICC-2)	
Patients with moderate-to-severe ARDS should be managed with invasive ventilation. Following only a brief, time-limited trial of HFNC or NIV — no longer than 4 h — stratify ARDS severity using the SpO₂/FiO₂ (S/F) ratio, calibrated when SpO₂ is between 90 and 97% [17] Moderate–severe ARDS: an oxygenation index/S-F ratio threshold consistent with severe disease is an absolute contraindication to NIV — proceed to intubation	
Absolute contraindications — do ***not*** start NIV	Relative contraindications — proceed with close monitoring
Cardiorespiratory arrest Depressed consciousness: GCS < 10 or absent gag/cough reflex Compromised airway: severe facial trauma or burns Haemodynamic instability: cardiogenic shock requiring inotropes Severe ARDS (PALICC-2 criteria)	Undrained pneumothorax Multi-organ failure Significant agitation or inability to cooperate Copious or thick secretions that cannot be cleared Recent upper GI or airway surgery

Inappropriate patient selection is the leading cause of delayed intubation and poor outcomes. Ask: If this child deteriorates on NIV, can we intubate quickly and safely here?

**Table 3:** ICEMAN framework — equipment (interface selection, checklist, and skin protection)

Interface options	Equipment checklist and skin protection
Nasal cannula: HFNC; simple, excellent tolerability, allows speech and feeding Total face mask: reduces leaks; fewer pressure sores and conjunctivitis; improves intubation rates Oronasal (full-face) mask: covers nose and mouth; needed for significant mouth leak or high pressures Nasal mask: first choice CPAP/BiPAP when child can close mouth; home ventilation, small infants Nasal pillows: older children/adolescents with claustrophobia Nasopharyngeal prong: young infants where mask sizing is impractical Helmet: craniofacial deformities; good evidence in hypoxaemic failure	Interface kit: Hydrocolloid dressings (apply prophylactically to nasal bridge) Correctly sized interface and harness Ventilator with paediatric circuit Heated humidifier and tubing Accessories: chin strap, spacers Fitting rules: Two fingers must fit beneath headgear straps — overtightening is the commonest cause of pressure injury Check for audible leak before increasing pressures In chronic users: alternate interfaces (pillows by day, nasal mask at night) Review at every clinic visit — sustained midface pressure causes midface hypoplasia

Evidence note: Pressure injuries affect 20–50% of children on long-term positive airway pressure [26], occurring most commonly at the nasal bridge and cheeks. Excessive mask leak is a significant independent risk factor for NIV failure

**Table 4:** ICEMAN framework — mode and settings (starting and titrating NIV, including age-specific considerations)

CPAP	BiPAP
Starting pressure: 5–6 cmH₂O Titrate: increase by 1–2 cmH₂O increments based on clinical response Maximum: 8–10 cmH₂O in children FiO₂: titrate to maintain SpO₂ 92–97% Use for: type 1 respiratory failure, obstructive sleep apnoea, post-extubation support	IPAP: start 10–12 cmH₂O; titrate up in 2 cmH₂O steps; maximum 18–20 cmH₂O EPAP: start 4–5 cmH₂O; increase if oxygenation poor despite adequate FiO₂; maximum 8–10 cmH₂O Back-up rate: set below natural rate (5–10 bpm below) for most; at natural rate for NMD or apnoeas FiO₂: titrate to SpO₂ 92–97% Use for: type 2 failure, hypercapnia, neuromuscular disease, post-extubation high risk
Age < 3 months/neonates	Age ≥ 3 months
Nasopharyngeal prong or nasal mask preferred Lower starting pressures: CPAP 4–5 cmH₂O; IPAP 8–10 cmH₂O; EPAP 3–4 cmH₂O Nasogastric tube for gastric decompression — aerophagy is common and impairs diaphragmatic excursion Heated humidification essential	Standard interface sizing by age/weight Low-dose dexmedetomidine may improve tolerance in young toddlers (SEDANIV trial, 2024; optimal agent/dose not yet established) Consider nasogastric tube if prolonged NIV anticipated BiPAP preferred over CPAP alone in type 2 failure or rising CO₂

**Table 5:** ICEMAN framework — assessment/monitoring and next steps (failure, weaning, escalation)

Minimum monitoring parameters	Signs of a good response (at 1–2 h)
Continuous SpO₂ and HR: mandatory SpO₂/FiO₂ ratio: surrogate for P/F ratio — target > 200 Respiratory rate: document every 30–60 min initially Work of breathing: assess at 0, 1, 2 h then 4-hourly Blood gas: at initiation and at key decision points; respiratory acidosis is a risk factor for failure TcCO₂: invaluable where available; removes need for repeated blood gas sampling Mask leak: excessive leak reduces delivered pressure and predicts failure Asynchrony: identify and correct promptly	Respiratory rate falling by ≥ 10 breaths/min Heart rate falling by ≥ 10 bpm Decreasing recession and accessory muscle use Stable or improving SpO₂ on same or lower FiO₂ SpO₂/FiO₂ ratio > 200 Improving or stabilising CO₂ on blood gas or TcCO₂ Child settles, stops fighting interface, appears more comfortable
Red flags — escalate immediately	Weaning protocol
Rising FiO₂: SpO₂ < 92% on FiO₂ > 0.6 despite optimised NIV Worsening hypercapnia: pH < 7.25 despite adequate BiPAP No improvement at 1–6 h: worsening or persistent increased work of breathing New contraindication: shock requiring inotropes; severe ARDS Declining consciousness: inability to protect airway Visible exhaustion: child can no longer sustain respiratory effort even with support	Weaning sequence: Wean FiO₂ first, then gradually reduce flow or pressure Do not wean precipitously — abrupt removal in bronchiolitis frequently causes rapid deterioration Allow interface breaks for comfort and feeding before full weaning trials Long-term NIV monitoring: Annual polysomnography or overnight TcCO₂ with oximetry Device download review at every clinic visit Regular interface and skin assessment

Note on NIV failure vs escalation decision: intubation necessitated by a new contraindication (e.g. haemodynamic collapse) should not be classified as NIV failure per se — the intervention appropriately achieved its bridge function until escalation became mandatory

Delayed intubation after NIV failure is independently associated with higher mortality. NIV is a bridge to recovery, intubation rates are approximately 18% in PICU settings, and red flags demand immediate action. Machine-learning models are also emerging to support earlier, more objective prediction of NIV failure in PICU populations [27]. Intubation necessitated by a new contraindication, such as haemodynamic collapse, should not itself be classified as NIV failure.

## Special populations and practical points

### The young infant

Infants under 6 months are obligate nasal breathers and tolerate HFNC well, but CPAP and BiPAP are often technically difficult to use due to limited oronasal mask sizes; total face masks have, however, shown good results in this population [28]. Nasopharyngeal CPAP, delivered via a short nasal tube, is a practical alternative for very young infants in low- and middle-income countries (LMIC). Gastric distension from aerophagy is common; as an institutional practice rather than a formally evidence-based protocol, we routinely insert a nasogastric tube for decompression in infants receiving CPAP or BiPAP for more than a few hours.

### The agitated or uncooperative child

In the FIRST-ABC Step-Up trial, the multicentre UK RCT comparing HFNC and CPAP as first-line respiratory support in the paediatric critical care unit, patient discomfort was the main reason for switching children from CPAP to an alternative modality [29], underscoring how poorly tolerated an interface can drive treatment failure independent of the underlying disease. In older children, invest time in preparation for mask application: explain what is happening at an age-appropriate level, include parents in holding the interface gently before it is applied formally, and use distraction with screens or toys. We can use sedation that does not cause respiratory depression in a child who will not cooperate with NIV. The multicentre Spanish SEDANIV study, published in 2024, found sedation was commonly used during paediatric NIV and was associated with improved tolerance, although the optimal agents and protocols remain to be established [30].

### The child on long-term NIV presenting acutely

We see these children on the ward during intercurrent illness, and it is important to know what to do. Do not discontinue NIV in a child who is NIV-dependent without specialist advice, as this can precipitate rapid decompensation. Obtain their NIV prescription, including settings, interface type, and daily use hours; download device data if possible; and contact the specialist unit early. Home NIV settings are optimised for stable baseline function, not for acute infection, so if they are no longer adequate for the child’s acute respiratory needs, escalate appropriately changing the interface to an oronasal mask or total face mask is often needed for adequate pressurisation.

### Resource-constrained settings

NIV is not just for tertiary centres. During the 2022–2023 respiratory virus surge, several centres implemented HFNC protocols in paediatric observation units and general wards to address PICU bed shortages while maintaining acceptable safety outcomes [31, 32]. Evidence from South Africa shows that HFNC and CPAP can be safely initiated at the district hospital level by non-specialist teams, with low complication rates during interfacility transfers [33]. The requirements are simple: well-trained staff, appropriate monitoring, a reliable oxygen supply, and a clear plan for escalation to a higher level of care when needed. We strongly advocate for the global dissemination of NIV skills at the generalist level with structured training of the medical and nursing teams.

## Conclusions

This review opened with the depiction of a child experiencing respiratory distress in the early hours, underscoring the urgency and complexity of such clinical scenarios. Every child in these circumstances must be cared for by a paediatric team proficient in selecting the most appropriate NRS or NIV option, applying interfaces correctly, monitoring for clinical progression, and identifying when intervention must be escalated. Non-invasive ventilation is based on well-established physiological principles and demands meticulous clinical judgement. Safe delivery of NIV outside the PICU also depends on the minimum structural conditions described in Sect. "Resource-constrained settings", i.e. continuous monitoring, staff experienced in recognising deterioration early, a genuine ability to intubate promptly if needed, and trained nursing support. Using the ICEMAN framework enables practitioners to initiate and manage NIV in a structured and systematic way, enhancing patient safety and optimising outcomes. Incorporating this approach into everyday paediatric practice helps to close existing gaps in care and provide better support for children with respiratory failure.

## Data Availability

No datasets were generated or analysed during the current study.

## Abbreviations

ARDS: Acute respiratory distress syndrome
ARF: Acute respiratory failure
AT: Adenotonsillectomy
BiPAP: Bilevel positive airway pressure
BROSJOD: Bronchiolitis Score of Sant Joan de Déu
CLD: Chronic lung disease
CPAP: Continuous positive airway pressure
DMD: Duchenne muscular dystrophy
EPAP: Expiratory positive airway pressure
FRC: Functional residual capacity
HDU: High dependency unit
HFNC: High-flow nasal cannula
ICEMAN: Indications, Contraindications, Equipment, Mode and settings, Assessment, Next steps
IPAP: Inspiratory positive airway pressure
LMIC: Low- and middle-income countries
M-WCAS: Modified Wood-Downes Clinical Asthma Score
NIV: Non-invasive ventilation
NMD: Neuromuscular disease
NRS: Non-invasive respiratory support
OSA: Obstructive sleep apnoea
PALICC-2: Second Pediatric Acute Lung Injury Consensus Conference
PARDS: Paediatric acute respiratory distress syndrome
PEEP: Positive end-expiratory pressure
PICU: Paediatric intensive care unit
RF: Respiratory failure
SMA: Spinal muscular atrophy
WOB: Work of breathing
